# Abdominal hernias in cirrhotic patients: Surgery or conservative treatment? Results of a prospective cohort study in a high volume center: Cohort study

**DOI:** 10.1016/j.amsu.2019.11.009

**Published:** 2019-11-22

**Authors:** Rafael Soares Pinheiro, Wellington Andraus, Daniel Reis Waisberg, Lucas Souto Nacif, Liliana Ducatti, Vinicius Rocha-Santos, Márcio A. Diniz, Rubens Macedo Arantes, Jan Lerut, Luiz Augusto Carneiro D'Albuquerque

**Affiliations:** a Digestive Organs Transplant Unit, Department of Gastroenterology, University of Sao Paulo School of Medicine, Sao Paulo, Brazil; b Biostatistics and Bioinformatics Research Center, Department of Medicine, Cedars Sinai Medical Center, Los Angeles, United States; c Starzl Unit of Abdominal Transplantation, University Hospital of Saint Luc, Université Catholique Louvain, Brussels, Belgium

**Keywords:** Liver cirrhosis, Hernia, Ascites, Abdominal wall/surgery, Survival analysis

## Abstract

**Background:**

Surgical treatment of abdominal hernias in cirrhotics is often delayed due to the higher morbidity and mortality associated with the underlying liver disease. Some patients are followed conservatively and only operated on when complications occur (“wait and see” approach). The aim of this study is to compare outcomes of cirrhotic patients undergoing conservative non-operative care or elective hernia repair.

**Methods:**

A prospective observational study including 246 cirrhotic patients with abdominal hernia was carried out. Patients were given the option to select their treatment: elective hernia repair or conservative non-operative care. Demographics, characteristics of underlying liver disease, type of hernia, complications and mortality were analyzed. During follow-up of patients who opted for the “wait and see” approach, emergency hernia repair was performed in case of hernia complications.

**Results:**

Elective hernia repair was performed in 57 patients and 189 patients were kept in conservative care, of which 43 (22.7%) developed complications that required emergency hernia repair. Elective surgery provided better five-years survival than conservative care (80% vs. 62%; p = 0.012). Multivariate analysis identified multiples hernias [Hazards Ratio (HR):6.7, p < 0.001] and clinical follow-up group (HR 3.62, p = 0.005) as risk factors for mortality. Among patients undergoing surgical treatment, multivariate analysis revealed MELD>11 (HR 7.8; p = 0.011) and emergency hernia repair (HR 5.35; p = 0.005) as independent risk factors for 30-day mortality.

**Conclusions:**

Elective hernia repair offers an acceptable morbidity and ensures longer survival. “Wait and see” approach jeopardizes cirrhotic patients and should be avoided, given the higher incidence of emergency surgery due to hernia complications.

## Highlights

• Prospective study comparing “wait and see” approach with elective surgical repair of abdominal hernias in cirrhotic patients.
• About 22.7% (n = 43) of patients under conservative treatment developed hernia complications requiring emergency hernia repair.
• Five-year survival was higher in elective repair group than in conservative treatment (80% vs. 62%; p = 0.012).
• Among patients undergoing hernia repair MELD>11 and emergency surgery were independent risk factors for 30-day mortality.
• “Wait and see” approach jeopardizes cirrhotic patients, as a high incidence of emergency surgery negatively impact survival.

## 1 Introduction

The incidence of umbilical hernia in cirrhotics surpasses 20% [1] in compensated disease [1] and 40% in patients with ascites [2]. Several risk factors explain this high incidence, such as recanalization of umbilical vein, sarcopenia and increased intra-abdominal pressure caused by ascites [3]. Compared to patients with no liver disease, complication rate is higher due to the development of pressure ulcers, skin rupture, ascites leak and bacterial peritonitis [2,4]. The higher intra-abdominal pressure also explains the development of large umbilical sacs and massive inguinal hernias that often reach the scrotum [5] and are responsible for reduced mobility and impaired quality of life [6].

The most appropriate treatment for many patients with abdominal hernias and advanced liver disease would be the concomitant correction of the anatomical defect and the underlying liver disease with liver transplantation (LT) [7]. Nevertheless, the number of available organs is scarce, resulting in a steadily growing waitlist.

In the past, the reported mortality rate of cirrhotic patients undergoing abdominal hernia repair was unacceptably elevated, reaching 16–31% [8,9]. Recurrence was also documented to be as high as 60% [9,10]. Owing to the fear of perioperative complications and hepatic decompensation with elective hernia repair, the “wait and see” approach was then implemented in many centers, in which most patients are followed conservatively and only operated on when complications occur [4,11]. This concept is still present nowadays and explains why a considerable amount of abdominal wall hernias in cirrhotic patients remain untreated for several years. More recent data, however, suggest that the risks associated with elective hernia repair are in fact much lower than historically reported [12,13]. Most importantly, emergent hernia repair, when compared to elective cases, is related to much higher morbidity and mortality rates. Delayed surgical treatment may therefore expose patients to a worse prognosis, compared to elective hernia repair. Decision-making on daily practice, nonetheless, remains obscure, since there are no prospective randomized studies to determine prognostic factors to accurately select cirrhotic patients for elective hernia repair or conservative treatment [14]. Most data derive from cases series and retrospective cohort studies.

The aim of this prospective study is to compare outcomes of cirrhotic patients undergoing conservative non-operative care (“wait and see” approach) or elective hernia repair.

## 2 Methods

The study included cirrhotic patients with abdominal hernias followed at the Department of Liver Transplantation of the University of São Paulo Medical School. It was approved by the Institutional Review Board of University of Sao Paulo School of Medicine and registered in ClinicsTrials.gov (NCT02787772). All patients provided informed consent. The study protocol followed the STROCSS criteria, available elsewhere [15].

A prospective cohort study was carried out from January 2009 to November 2014. After being informed about the lack of medical literature consensus concerning hernia repair in the context of cirrhosis, patients were given the option to select their treatment: elective hernia repair or conservative non-operative care (“wait and see” approach). The advantages and risks of each option were extensively discussed before reaching a decision. Patients who chose conservative treatment had clinical follow up similar to cirrhotic patients without hernia. In case of hernia complications during “wait and see” approach - such as incarceration, strangulated bowel, necrotic skin and ascites leakage - patients were evaluated and had emergency hernia repair if indicated.

Abdominal wall hernias were diagnosed by physical examination. Ultrasound or abdominal computed tomography scans were used in cases of diagnostic doubt or for surgical planning. Ascites was managed individually when present, with sodium-restricted diet, diuretic medications, therapeutic paracentesis or transjugular intrahepatic portosystemic shunt (TIPS). Patients with indication of liver transplantation were listed during the study or already in the waitlist previously to it.

All hernias repairs were performed open and under general anesthesia. Intravenous antibiotics were prophylactically administered intraoperatively as well as albumin when ascitic fluid was drained (10 g/L, up to 100g). For umbilical and incisional hernias, an onlay polypropylene mesh was used following the correction of anatomical defect. A continuous suction drain was left in subcutaneous in order to drain and quantify possible bleeding or ascitic fluid leakage. Inguinal hernias were repaired according to the Lichtenstein technique. Patients presenting with multiples hernias underwent simultaneous repair of all sites.

The following variables were studied: age, sex, etiology of liver disease, Child- Turcotte-Pugh (CTP) classification, Model of End-Stage Liver Disease (MELD) score, degree of ascites, presence of diabetes mellitus and chronic kidney disease requiring renal replacement therapy, abdominal hernia type (umbilical, inguinal or incisional) and inclusion in liver waitlist. Multiple hernias were defined as more than one hernia type in a same patient. For patients who underwent surgery, number and grade of post-operative complications were registered following Clavien's classification [16]. Postoperative need of paracentesis and length of hospital stay were also recorded. Postoperative renal dysfunction was defined as a 1.5 times increase in creatinine serum levels, requiring albumin, terlipressin or hemodialysis. Early post-operative mortality was considered as any event occurring within 30-days after surgery. Outcomes of the study were death, hernia recurrence or liver transplantation.

## 3 Statistical analysis

Quantitative variables were analyzed using t or Mann-Whitney test conditioned by the assumptions of normality and homogeneity of variances, respectively, verified by Anderson-Darling and Levene tests. For qualitative variables, Fisher's test was used.

Survival was defined as the period from the diagnosis to either death or loss of follow-up. Survival curves were made following the Kaplan-Meier method. For the quantitative variables, the cutoff point that best differentiated survival between groups was identified from maximizing the log-rank statistic, as proposed by Lausen et al. [17]. The simple Cox regression was used to estimate the hazard ratio death, with their respective 95% confidence interval, and the proportionality assumption was verified by Schoenfeld residuals [18].

In a second step, a multiple regression analysis was performed considering the variables with p values less than 0.10 in the simple analysis. The significance level for the analysis was 0.05. All statistical analyses were performed with statistical package R, version 3.1.2 (R Foundation for Statistical Computing; 2016).

## 4 Results

**Fig. 1:**
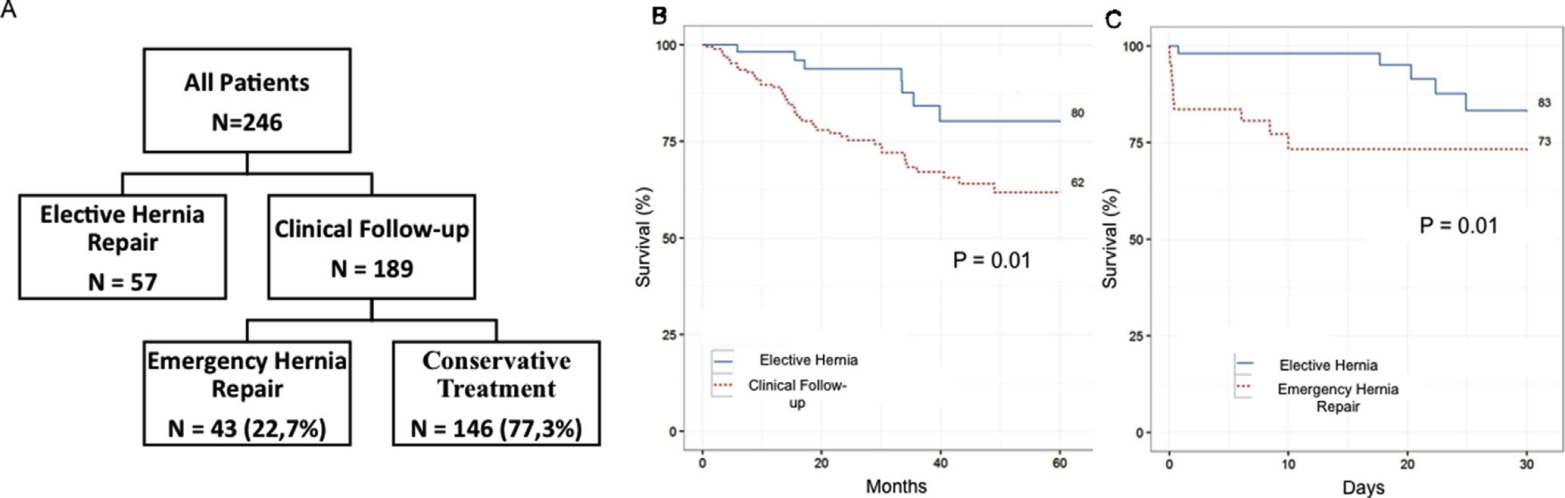
**A:** Patient allocation during the study. **B:** Long-term survival of elective hernia repair and clinical follow-up groups. **C:** Postoperative 30-day survival after elective or emergency hernia repair surgery.

A total of 246 patients were included in the study: 57 who underwent elective hernia repair and 189 patients who were followed-up. Of these, 43 patients underwent emergency hernia repair due local complications (Fig. 1A). Umbilical hernia was the most common hernia type, followed by multiple sites hernias (Table 1).

**Table 1:** Distribution of hernia types in relation to treatment option.

Type of Hernia	Elective Repair N = 57	Clinical follow-up N = 189	
		Emergency Surgery N = 43	Conservative Treatment N = 146
Umbilical hernia	28 (49.1%)	33 (76.7%)	72 (49.3%)
Groin hernia	10 (17.5%)	2 (4.6%)	25 (17.1%)
Incisional hernia	1 (1.8%)	1 (2.3%)	9 (6.2%)
Multiple hernias	18 (31.6%)	7 (16.4%)	40 (27.4%)

Median follow-up time for the entire population was 2.04 years (8–2241 days). Median follow-up time for patients who underwent elective hernia repair and for those who opted for conservative non-operative care was 2.3 and 2.12 years, respectively. All patients were followed for 6 months at least. Follow-up was lost in 13 patients who opted for conservative treatment.

Patients' characteristics are shown in Table 2. Mean patients’ age was 65.52 ± 11.06 years and median MELD was 12 [[6], [7], [8], [9], [10], [11], [12], [13], [14], [15], [16], [17], [18], [19], [20], [21], [22], [23], [24]]. All variables were similar between the two groups, except for number of patients in LT waiting list, which was higher in the elective hernia repair groups. Eighty-one patients were in the LT waiting list at the beginning of the study and 27 eventually underwent transplantation. Five of them were transplanted during early hernia repair postoperative period due to MELD increase.

**Table 2:** Patients’ characteristics.

Variable	Clinical Follow- up N = 189	Elective Hernia Repair N = 57	p
Male	136 (71.9%)	46 (80.7%)	0.229
Age > 60 years	74 (39.1%)	18 (31.5%)	0.350
Alcoholic Cirrhosis	74 (39.1%)	22 (38.6%)	0.980
Hepatitis C Virus infection	61 (32.2%)	14 (24.5%)	0.325
CTP A	62 (32.8%)	17 (29.8%)	0.747
CTP B	104 (55%)	32 (56.1%)	0.321
CTP C	19 (10%)	8 (14%)	0.465
MELD >11	99 (52.4%)	35 (61.4%)	0.291
Mild Ascites	48 (25.4%)	19 (33.3%)	0.239
Tense Ascites	35 (18.5%)	6 (10.5%)	0.222
Umbilical Hernia	144 (76.2%)	43 (75.4%)	1
Unilateral Groin Hernia	62 (32.85)	21 (36.8%)	0.632
Bilateral Groin Hernia	12 (6.3%)	7 (12.2%)	0.159
Incisional Hernia	19 (10%)	2 (3.5%)	0.176
Multiples Hernias^a^	47 (24.9%)	18 (31.6%)	0.233
Number of patients in LT Waiting List	52 (27.5%)	29 (50.8%)	**0.002**
Referred Pain	136 (71.9%)	46 (80.7%)	0.230
Diabetes Mellitus	38 (20.1%)	10 (17.5%)	0.849
Chronic Kidney Disease	3 (1.6%)	2 (3.5%)	0.328

CTP, Child-Pugh Score. MELD, Model of End-Stage Liver Disea

a Including bilateral groin hernia and more than one hernia site in a same patient.

Complications leading to emergency hernia repair were ascites leakage due skin rupture in the hernia site in 28 patients (65%), incarceration in 7 patients (16%), small bowel strangulation 5 patients (12%), and extensive skin necrosis or ulceration 3 patients (7%). Four cases (2 bowel strangulation and 2 skin ruptures) occurred in groin hernias, all others complications occurred in umbilical hernias (90.7%).

One, three and five-years actuarial overall survivals for the entire population were 91.1% (Confidence Interval [CI] 95% 87.5–94.8%), 71.2% (CI 95% 64.4–78.6%) and 66.2% (CI 95% 58.5–74.9%), respectively. Patients in the clinical follow-up showed lower survival rate than patients in the elective hernia repair group (Fig. 1B). Univariate cox regression test identified age >60 years, CTP, MELD >11, tense ascites, being in the LT waiting list, multiples hernias and clinical follow-up group as risk factors for mortality (Table 3). Multivariate analysis confirmed clinical follow-up group as a factor of worse prognosis.

**Table 3:** Univariate and Multivariate Cox regression for long-term mortality risk factors.

Variable	HR	CI 95%		P value
Univariate Regression				
Male	0.61	0.35	1.07	0.422
Age >60 years	1.93	1.14	3.25	**0.014**
CTP B	1.97	1	3.88	**0.052**
CTP C	5.92	2.55	13.75	**<0.001**
MELD >11	2.38	1.33	4.26	0.003*
Tense Ascites	2.52	1.20	5.29	0.015
Umbilical Hernia	1.87	0.92	3.82	0.086
Unilateral groin Hernia	1.21	0.70	2.09	0.496
Bilateral Groin Hernia	0.83	0.29	2.37	0.733
Incisional Hernia	0.92	0.33	2.53	0.865
Multiple hernias	5.97	3.49	10.22	**<0.001**
Waiting List for LT	1.97	1.16	3.33	**0.011**
Diabetes Mellitus	1.52	0.84	2.75	0.165
Clinical Follow up	2.62	1.19	5.79	**0.017**
Multivariate Regression				
Multiple hernias	6.70	3.65	12.31	<0.001
Clinical Follow-Up	3.62	1.48	8.82	0.005*

CTP, Child-Pugh Score. HR: Hazard Ratio. MELD, Model of End-Stage Liver Disease. LT, Liver Transplantation.

### 4.1 Hernia repair: elective versus emergency surgery

Characteristics of patients operated on electively and in the emergency setting are detailed in Table 4. Mean age was 54.39 ± 10.94 years and median MELD was 12.5, ranging from 6 to 23. More patients with tense ascites and less CTP A patients were operated on urgently. A total of 7 patients underwent TIPS, 3 electively and 4 after skin rupture in hernia site. Length of hospital stay was longer in patients undergoing emergency surgery than those operated on electively (20.65 ± 17.7 days and 6.84 ± 6.21 days, respectively, p = 0.001) as well as intensive care unit stay (4.67 ± 7.09 days and 6.84 ± 6.21 days, respectively, p = 0.001).

**Table 4:** Characteristics of patients who underwent surgical hernia repair.

Variable	Elective Surgery N = 57	Emergency Surgery N = 43	p
Male	46 (80.7%)	29 (67.4%)	0.163
Age >60 years	18 (31.5%)	14 (32.5%)	1
Alcoholic Cirrhosis	22 (38.6%)	20 (46.5%)	0.539
Hepatitis C	14 (24.5%)	9 (20.9%)	0.811
CTP A	17 (29.8%)	3 (7%)	**0.005**
CTP B	32 (56.1%)	30 (70%)	0.212
CTP C	8 (14%)	10 (23%)	0.295
MELD >11	35 (61.4%)	28 (65.1%)	0.835
Mild Ascites	19 (33.3%)	19 (44.2%)	0.302
Tense Ascites	6 (10.5%)	19 (44.2%)	**0.0002**
LT Waiting list	29 (50.8%)	17 (39.5%)	0.313
Diabetes Mellitus	10 (17.2%)	3 (6.98%)	0.144
Renal Failure	2 (3.5%)	2 (4.6%)	1
Umbilical Hernia	43 (75.4%)	39 (90.7%)	0.066
Unilateral Groin Hernia	21 (36.8%)	8 (18.6%)	0.074
Bilateral Groin Hernia	7 (12.2%)	1 (2.3%)	0.462
Incisional Hernia	2 (3.5%)	2 (4.6%)	1
Multiples hernias	18 (31.6%)	7 (16.3%)	0.067
Postoperative Complications			
Any Complication	20 (35.1%)	35 (81.4%)	**<0.001**
Major Complication (Clavien grade III or higher)	8 (14%)	27 (62.8%)	**<0.001**
Postoperative paracentesis	6 (10.5%)	27 (62.8%)	**<0.001**
Postoperative Renal Dysfunction	13 (22.8%)	31 (72.1%)	**<0.001**
Superficial Wound Dehiscence	6 (10.5%)	6 (13.9%)	0.758
Infection Complications^a^	7 (12.2%)	22 (51.15%)	**<0.001**

CTP, Child-Pugh Score. MELD, Model of End-Stage Liver Disease.

a Surgical wound infection or secondary bacterial peritonitis.

Postoperative complications included postoperative need of paracentesis; postoperative renal dysfunction; surgical wound dehiscence, hematoma or infection; scrotum hematoma, upper digestive hemorrhage and secondary bacterial peritonitis. They were more frequent and more severe in patients undergoing emergency surgery (Table 4). Thirty-day mortality was significantly higher in those patients as well (p = 0.01) (Fig. 1C). Univariate and multivariate analyses identified emergency hernia repair and MELD >11 as an independent risk factors for 30-day mortality (Table 5).

**Table 5:** Univariate and Multivariate Cox regression for 30-day mortality of patients who underwent surgical hernia repair.

Variable	HR		CI 95%		p
Emergency Surgery	3.77		1.27	11.23	0.017
MELD >11	5.47		1.22	24.52	**0.026**
Multiples hernias	2.49		0.88	7.01	0.085
Male	0.49		0.18	1.39	0.181
Groin hernia	0.54		0.15	1.97	0.350
Waiting List for LT	0.66		0.23	1.94	0.453
Tense Ascites	1.88		0.34	10.41	0.468
Diabetes Mellitus	0.47		0.06	3.6	0.469
Umbilical Hernia	1.73		0.39	7.7	0.472
Mild Ascites	1.45		0.29	7.17	0.652
Age >60 years	0.85		0.27	2.68	0.782
Multivariable Regression					
Emergency Surgery	5.35		1.64	17.45	**0.005**
MELD >11	7.8		1.6	38.98	**0.011**
Multiples Hernias	2.77	0.97		7.91	0.057

CTP, Child-Pugh Score. HR: Hazard Ratio. MELD, Model of End-Stage Liver Disease. LT, Liver Transplantation.

## 5 Discussion

This study encompasses a prosprective trial that compared elective hernia repair and conservative non-operative care (“wait and see” approach) in a large sample of cirrhotic patients with abdominal hernias. Our main result shows that conservative treatment is independently associated with decreased survival, especially due to the greater morbidity and mortality of emergency operations performed when complications occur. Emergency surgery due to hernia local complications took place in 22.7% of patients under “wait and see” approach.

Over the last decade, elective hernia surgery has been increasingly advocated for cirrhotic patients [2,12,19,20]. Modern surgical technique and perioperative care have been reported to improve morbity and mortality rates, justifying early repair before complications arise. Emergency operations have been reported as preditors for worse outcome as well as higher CTP and MELD scores [13,21,22]. Interestingly, morbidity increass 3% for each additional MELD point above 15 [13]. Morbidity and mortality rates for elective and emergency procedures ranges from 7.4 to 27%/3.4–3.7% and 44–60% and 10–29% [21,22]. Moreover, non-operative treatment for abdominal hernias also impairs quality of life [6,23]. Delaying surgical treatment in CTP C patients may be hazardous, as mortality may reach 57% in emergency repair [24].

Morbidity and mortality rates vary according to the degree of liver disease. In Hew et al. series, they were or CTP A patients, 18.8% and 0% for CTP B and 66.7% and 4.2% for CTP C, respectively [25]. This data shows that although complications become more prevalent as liver disease advances, the outcomes are still satisfactory, and thus CTP score itself should not be a sole reason to preclude elective hernia repair. As severity of cirrhosis increases, patients are less likely to be selected for elective surgery. Among patients with CTP class A, B and C, the frequencies of elective hernia repair were 80, 52 and 17% in a classical series reported by Belguiti et al. [10]. In a more recent study, the incidence of CTP C patients was 16% in the elective group, while it was 90% in those who underwent emergency surgery [26].

Ascites and umbilical hernias in special represent a serious combination that might lead to more frequent recurrences and emergency operations. Several series have reported increased recurrence rates reaching up to 71% [10,27]. In our study, complicated umbilical hernias were responsible for 9 out of 10 emergency procedures and almost half of patients operated on urgently presented tense ascites (44.2%). The need of postoperative paracenthesis was also much higher in patients operated on urgently than electively (62.8% *versus* 10.5%, p < 0.001). The control of ascites is hence critical for the success of umbilical hernia repair and it may be achieved preoperatively with diuretics and sodium restriction. If refractory, it may be controlled either non-invasively by routine paracentesis or using TIPS [28,29]. Surgical shunts are exceptionally used currently. Hernia recurrence is also lowered by using prosthetic mesh. This might explain the low recurrence rate found in our study (2%), similar to the ones reported in others series (1,7 to 2.7%) [25,26].

Upfront elective hernia repair cannot be advocated for all cirrhotics. Patients with decompensated liver disease should be medically managed preoperatively, especially regarding renal dysfunction, coagulation disorders, and adequate ascites control. For patients with spontaneous umbilical rupture due to refractory ascites, preoperative TIPS and semi-elective repair, when feasible, may improve outcome [28,29]. Albeit important, optimal ascites control must not preclude elective repair in high risk patients for hernia complications, such as those with thin bright skin, skin ulcerations or extremely symptomatic. The perspective of liver transplantation should be considered as well. If LT is expected to occur briefly, hernia repair may be delayed if the risk of local complication is low. In our study, 32.92% of patients were at LT waitlist and one third of these underwent LT during the study. This demonstrates the recommendation that patients with advanced cirrhosis and abdominal wall hernias should be preferentially treated in specialized centers who also offer transplantation [21,25]. Hernia diagnoses in patients with portal hypertension may be tricky. The presence of collateral vein in the spermatic cord can mimic a groin hernia [30]; and hernia sacs present occasionally in unusual locations such as the perineum [31,32]. The availability of LT is also paramount, as probability of transplantation growths as the MELD increases, which commonly occurs in the postoperative period of hernia repair due to acute exacerbation of liver disease [33]. This was in fact the case of 18.5% of LT in in our series.

This is a prospective cohort study with a large number of cirrhotic patients with abdominal wall hernias, evaluating outcomes of elective hernia repair or conservative approach. Unfortunately, it is not without limitations. Groups formation was not randomized and could have been biased since it could be expected that sicker patients would not be candidates for elective hernia repair. Additionally, patients could opt for elective surgery at any point of the study and therefore patients could favor surgery as symptoms developed. Nevertheless, all variables were similar between groups, with exception of patients in LT waitlist. Regarding elective and emergent surgery analysis, there were more patients CTP A in the first group while there were more patients with tense ascites in the second. The main cause of emergency hernia repair was in fact rupture of the skin with ascites leak at umbilical hernias, which is less frequent in CTP A patients. Similar studies from others centers are therefore advised to validate our results.

In conclusion, elective abdominal hernia repair in cirrhotics offers acceptable morbidity and ensures longer survival than conservative treatment. “Wait and see” approach jeopardizes cirrhotic patients and should therefore be avoided, given the higher incidence of emergency surgery due to hernia complications, which are associated with increased postoperative morbidity and mortality. Hernia repair in cirrhotic patients should be preferably done in specialized centers experienced in managing advanced liver disease, where liver transplantation is readily available.

## Ethical approval

This study was approved by the Institutional Review Board of University of Sao Paulo School of Medicine (Cappesq number 0937/09).

## Sources of funding

No source of funding is related to this study.

## Author contribution

RSP: Study concept and design, data collection, data analysis, manuscript preparation.

WA: Study concept and design, data analysis, critical review of manuscript.

DRW: data collection, data analysis, manuscript preparation.

LSN: data collection, data analysis, manuscript preparation.

LDL: data collection, data analysis.

VRS: data collection, data analysis.

MAD: data analysis.

RMA: data collection, data analysis.

JL: study concept and design, critical review of manuscript.

LACD: study concept and design, critical review of manuscript.

## Research registration number

Name of the registry: ClinicalTrials.gov.

Unique Identifying number or registration ID: NCT02787772.

Hyperlink to the registration (must be publicly accessible): https://clinicaltrials.gov/ct2/show/NCT02787772?id=NCT02787772&rank=1.

## Guarantor

The Guarantors of this study are Rafael Soares Pinheiro, Wellington Andraus and Luiz Augusto Carneiro D'Albuquerque.

## Consent

All patients involved in this study provided informed consent.

## Provenance and peer review

Not commissioned, externally peer reviewed.

## Declaration of competing interest

All authors declare no conflict of interest related to this study.

## Appendix A Supplementary data

the_strocss_statement_checklist the_strocss_statement_checklist
